# *Lactobacillus rhamnosus* GG Suppresses Meningitic *E. coli* K1 Penetration across Human Intestinal Epithelial Cells In Vitro and Protects Neonatal Rats against Experimental Hematogenous Meningitis

**DOI:** 10.1155/2009/647862

**Published:** 2008-11-24

**Authors:** Sheng-He Huang, Lina He, Yanhong Zhou, Chun-Hua Wu, Ambrose Jong

**Affiliations:** ^1^Department of Pediatrics, The Saban Research Institute of Childrens Hospital Los Angeles, University of Southern California, Los Angeles, CA 90027, USA; ^2^School of Life Science and Technology, HuaZhong University of Science and Technology, Wuhan, Hubei 430074, China

## Abstract

The purpose of this study was to examine prophylactic efficacy of probiotics in neonatal sepsis and meningitis caused by *E. coli* K1. The potential inhibitory effect of *Lactobacillus rhamnosus* GG (LGG) on meningitic *E. coli* K1 infection was examined by using (i) in vitro inhibition assays with E44 (a CSF isolate from a newborn baby with *E. coli* meningitis), and (ii) the neonatal rat model of *E. coli* sepsis and meningitis. The in vitro studies demonstrated that LGG blocked E44 adhesion, invasion, and transcytosis in a dose-dependent manner. A significant reduction in the levels of pathogen colonization, *E. coli* bacteremia, and meningitis was observed in the LGG-treated neonatal rats, as assessed by viable cultures, compared to the levels in the control group. In conclusion, probiotic LGG strongly suppresses meningitic *E. coli* pathogens in vitro and in vivo. The results support the use of probiotic strains such as LGG for prophylaxis of neonatal sepsis and meningitis.

## 1. Introduction

Bacterial sepsis and meningitis continue to be the most common serious infection in neonates [1–3]. Group B Streptococcus (GBS) and *E. coli* are the two most common bacterial pathogens causing neonatal sepsis and meningitis (NSM) [2, 3]. Invasive GBS disease emerged in the 1970s as a leading cause of newborn morbidity and mortality in the US [4]. Extensive studies demonstrated that intrapartum prophylaxis (IP) of GBS carriers and selective administration of antibiotics to neonates decrease newborn GBS infection by as much as 80 to 95% [4–7]. However, a major concern is whether the IP use of antibiotics affects the incidence and the resistance of early onsetneonatal infection with non-GBS pathogens [4–7]. Currently, the focus has been shifted to *E. coli*, which is a leading cause of infection among neonates, particularly among those of very low birth weight (VLBW) [8]. Although initially most multicenter reports showed stable rates of non-GBS early onset infection with IP for GBS, more recent studies challenge this conclusion, suggesting an increasing incidence of early onset *E. coli* infections in low birth weight and VLBW neonates and a rising frequency of ampicillin-resistant *E. coli* infections in preterm infants [9, 10]. Widespread antibiotic use (WAU), particularly with broad-spectrum antimicrobial agents, may result in a rising incidence of neonatal infections with antibiotic resistance, which is an ecological phenomenon stemming from the response of bacteria to antibiotics [11]. Antibiotic resistance has emerged as a major public health problem during the past decade [12]. WAU will certainly worsen the ongoing antimicrobial resistance crisis.

The development of microbial infections is determined by the nature of host-microbe relationships. As most microbes form a healthy symbiotic “superorganism” with the hosts, a holistic balance of this relationship is essential to our health [13]. This ecological balance is affected by environmental factors, which include the use of antibiotics, immunosuppressive therapy, irradiation, hygiene, and imbalance of nutrition. Some of the mentioned factors may contribute to a decline in the incidence of microbial stimulation that may dampen host defense and predispose us to infectious diseases [14, 15]. Therefore, the introduction of beneficial microorganisms such as probiotics into our body is a very attractive rationale for modulating the microbiota, improving the symbiotic homeostasis of the superorganism, and providing a microbial stimulus to the host immune system against pathogens including meningitic *E. coli* K1. As probiotics help to maintain ecological balance, the use of probiotics for the prophylaxis of early onset neonatal meningitic infections may overcome the major disadvantage of WAU, which disturbs the normal microbiota. Studies using LGG have demonstrated that atopic dermatitis of newborns can be prevented in 50% of cases if mothers take probiotics and neonates ingest LGG during the first 6 months of life [16]. Newborns fed with LGG-enriched formula grew better than those fed with the regular one [17]. LGG has been shown to decrease the frequency and duration of diarrhea caused by *E. coli* and other pathogens [18, 19]. However, it is unknown whether probiotics are effective in preventing NSM. In order to dissect this issue and develop probiotics as a better approach for the prophylaxis of NSM caused by meningitic pathogens including GBS and *E. coli* K1, prophylactic efficacy of LGG in NSM was tested in vitro and in vivo.

## 2. Materials and Methods

### 2.1. Bacterial Strains and Culture Conditions

The commercial probiotic strain used was *L. rhamnosus* GG (LGG) (ATCC 53103). The bacterial pathogen used was *E. coli* E44, a rifampin-resistant strain of a clinic isolate *E. coli* RS218 (O18:K1:H7) from the cerebrospinal fluid (CSF) of a newborn infant with meningitis [20]. LGG was grown in Rogosa SL broth (Difco) at 37°C for 18 hours. The culture was centrifuged (10 000 × *g* for 5 minutes at 4°C), and bacteria were suspended in cell culture medium. The final suspension was adjusted to obtain the appropriate concentration. E44 was grown at 37°C in Luria-Bertani (LB) or brain-heart infusion (BHI) broth with rifampin (100 *μ*g/mL) overnight. After centrifugation, bacteria were suspended in cell culture medium. The number of CFU was determined by plating serial 10-fold dilutions from bacterial suspensions on LB (for E44) or Rogosa SL (for LGG) agar plates. Plates were incubated at 37°C in a CO_2_ atmosphere overnight.

### 2.2. Cell Culture Model of Intestinal Epithelial Cell Line

Caco-2 cells were obtained from American Type Culture Collection (ATCC, Rockville, Md, USA) and used between passages 19 and 23 as older passages have shown to be less permissive to bacterial entry [21]. Caco-2 cells were grown in Eagle's Minimum Essential Medium supplemented with 20% heat-inactivated FCS, 1% Cellgro nonessential amino acids, 2 mM L-glutamine, 1 mM sodium pyruvate, 100 000 U/L penicillin, 100 mg/L streptomycin, and 2.5 mg/L Fungizone. The cells were incubated in a 5% CO_2_ atmosphere at 37°C in T-75 tissue culture flasks coated with rat-tail collagen. Cells used for the quantitative adhesion and invasion assays were seeded at 10^5^ cells per well, in a 24-well tissue culture plate coated with rat-tail collagen, and assays were performed at a minimum of 12 days postconfluence. At this time, the polarized monolayers exhibited dome formation, characteristic of transporting epithelia, and evidence of end-stage differentiation [22].

### 2.3. Adhesion and Invasion Assays

Bacterial inocula were prepared in experimental media without antibiotics (Ham's F12: Medium 199 1X Earl's Salts in a 1:1 ratio, 5% heat-inactivated fetal bovine serum, 1% sodium pyruvate, and 0.5% L-glutamine). Caco-2 cells were inoculated with 1 × 10^7^ bacteria per well, to give a multiplicity of infection of 100 and be incubated for 90 minutes at 37°C in 5% CO_2_ atmosphere to allow bacterial adhesion and entry. The adhesion assays were carried out as described previously [23]. To determine the total number of cell-associated bacteria, the cells were washed three times with medium and lysed with 100 *μ*l of 0.5% Triton X-100 for eight minutes followed immediately by the addition of 50 *μ*l of sterile water. The monolayers remained intact throughout the incubation and washing phases of the assay until lysis. This concentration of Triton X-100 did not affect bacterial viability for at least 30 minutes (data not shown). Samples were diluted and plated onto sheep blood agar plates to determine the number of colony forming units (CFUs) recovered from the lysed cells. The number of associated bacteria was determined after washing off the unbound bacteria. A percent adhesion was calculated by [100 × (number of intracellular bacteria recovered)/(number of bacteria inoculated)]. Each experiment was carried out in triplicate, and results presented are representative of the repeated assays.

For invasion assays, the same number (10^7^) of bacteria was added to confluent monolayers of Caco-2 with a multiplicity of infection of 100 [24]. The monolayers were incubated for 1.5 hours at 37°C to allow invasion to occur. The number of intracellular bacteria was determined after the extracellular bacteria were eliminated by incubation of the monolayers with the experimental medium containing gentamicin (100 *μ*g/mL) for 1 hour at 37°C. Results were expressed either as percent invasion [100 × (number of intracellular bacteria recovered)/(number of bacteria inoculated)] or relative invasion (percent invasion as compared to the invasion of the parent *E. coli* K1 strain).

### 2.4. Transcytosis Assay

Caco-2 cells were cultured on 6.5-mm diameter, collagen-coated Transwell polycarbonate cell culture inserts with a pore size of 3 *μ*m (Corning Costar Corp., Cambridge, Mass, USA) for at least 5 days as previously described [25, 26]. This in vitro model of the gut barrier allows separate access to the upper chamber (gut side) and lower chamber (blood side) and permits mimicking of NSM *E. coli* penetration across the gut barrier into the blood stream. Human epithelial cells are polarized and exhibit a transepithelial electric resistance (TEER) of at least 300 Ω/cm^2^ [27] as measured with an Endohm volt/ohm meter in conjunction with an Endohm chamber (World Precision Instruments, Fla, USA) as previously described [26]. On the morning of the assay, the Caco-2 monolayers were washed, experimental medium was added, 10^5^ E44 cells were added to the upper chamber (total volume, 200 *μ*L), and the monolayers were incubated at 37°C. At 2, 4, and 6 hours, samples of 30 *μ*L were taken from the lower chamber (an equivalent volume of medium was immediately added, maintaining a total bottom volume of 1 mL) and plated for CFU determination. The integrity of the Caco-2 monolayer was assessed by measuring TEER and horseradish peroxidase (HRP) permeability. The results were expressed as the percentage of initial inoculum transcytosed. The cell numbers were determined based on the viable-cell counts on the blood agar plates.

### 2.5. Competitive Exclusion Assays

Three different procedures (adhesion, invasion, and transcytosis) were used to assess exclusion of E44 strain by LGG. Exclusion was assessed by performing preinfection experiments in which cultured intestinal epithelial cells were first incubated with LGG. NSM strain E44 was added for further incubation. The numbers of strains adhering to, invading, or crossing the intestinal cells were determined as described above. For each assay, a minimum of three experiments was performed with successive passage of intestinal cells. To test the effects of LGG on *E. coli* K1 adhesion to and invasion of Caco-2, the cells were subcultured into 24-well tissue culture plates and then preincubated at 37°C with 1 × 10^7^ and 1 × 10^8^ CFU of LGG in the complete experimental medium for 3 hours. After incubation with LGG, 1 × 10^7^ CFU of E44 was added to the cultures followed by incubation at 37°C for 2 hours to allow adhesion and invasion to occur. Adhesion/invasion assays and result expressions were performed as described above. To examine effects of LGG on E44 translocation across human intestinal epithelial cell monolayers, the cell cultures were incubated at 37°C with 1 × 10^7^ to 1 × 10^8^ CFU of LGG for 3 hours. After incubation with LGG, 1 × 10^7^ CFU of E44 was added to the upper chamber of Transwell. The appearance of E44 in the bottom chamber was determined as described above.

### 2.6. Neonatal Rat Model of Hematogenous *E. coli* K1 Meningitis

The ability of *E. coli* strains E44 to colonize and cause NSM in vivo was examined in a neonatal rat model. All animal experiments were carried out with prior approval from the Animal Care Committee of Childrens Hospital Los Angeles Research Institute (Calif, USA). Pathogen-free Sprague Dawley rats with timed conception delivered pups on the seventh day after arrival. In order to determine the therapeutic efficacy of LGG, a pilot study with two groups (14 pups/group) of animals was carried out. The pups were pooled and randomly distributed into the experiment group (LGG) and control group (PBS). Pups on day 2 of life received oral LGG or PBS by feeding the pups using an FB Multiflex tip (from Fisher Scientific, Pa, USA). Daily dose of LGG was 10^10^ CFU/kg or 10^7^ CFU/g. Control rats received PBS only. At 5 days of age, all pups received 10^9^ CFU/pup of E44 by feeding the animals with the same delivery approach. Stool, blood, and CSF samples were taken for quantitative cultures at 48 hours after oral inoculation. Stool samples were obtained by aspirating rectal contents through 1cm of sterile plastic tubing (intramedic polyethylene tubing, outer diameter 0.61 mm) with a sterile tuberculin syringe. The stool aspirate and tubing were placed in 950 *μ*L of BHI broth and homogenized. This solution was plated onto sheep blood agar or grown in BHI broth. Intestinal colonization was defined as a positive stool culture, from either the agar plate or the overnight broth. Blood cultures were obtained in a sterile fashion from the right external jugular vein. Blood was diluted in BHI and plated onto sheep blood agar. Bacteremia was defined as a positive blood culture. CSF samples were obtained and cultured as described previously [28]. Meningitis was defined as positive CSF cultures.

### 2.7. Statistical Analysis

Data were analyzed as described previously [29]. Analysis of variance (ANOVA) and covariates followed by a multiple comparison test such as the Newmann-Keuls test were used to determine the statistical significance between the control and treatment groups; *P* < .05 was considered to be significant.

## 3. Results

### 3.1. Effects of Probiotics on Meningitic *E. coli* K1 (E44) Adhesion to and Invasion of Human Intestinal Epithelial Cells in vitro

**Figure 1:**
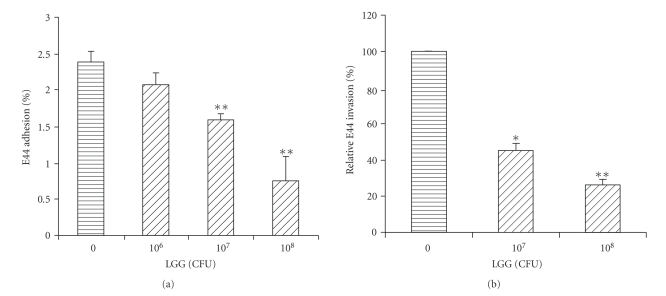
Effects of LGG on *E. coli* K1 adhesion to and invasion of Caco-2. Epithelial cells were incubated with various doses of *L. rhamnosus* for 3 hours before adding bacteria. Adhesion and invasion assays were carried out as described above. All values represent the means of triplicate determinations. The results were expressed as adhesion (Figure 1(a)) or invasion activities (Figure 1(b)) compared to that of the control without LGG. Error bars indicate standard deviations. *P < .05; **P < .01.

Caco-2 is used as an in vitro model for testing effects of LGG on meningitic *E. coli* adhesion to and invasion of the gut barrier since it has been one of the most relevant in vitro models for the studies of small intestinal epithelial cell differentiation and transport properties [22]. The ability of LGG to interfere with the adhesion of *E. coli* K1 to Caco-2 cells was examined by competitive exclusion/adhesion inhibition assays. In this study, Caco-2 cells were preincubated with different doses of LGG (10^6^ to 10^8^ CFU) before the addition of meningitic *E. coli* K1 strain E44. As shown in Figure 1(a), LGG was able to competitively inhibit E44 invasion of Caco-2 cells in a dose-dependent manner (*P* < .01). Effects of LGG on the invasive phenotype of strain E44 into Caco-2 cells were determined utilizing competitive exclusion/invasion inhibition assays. Caco-2 cells were preincubated with different doses of LGG (10^7^ to 10^8^ CFU) before addition of meningitic *E. coli* K1 strain E44. The intracellular *E. coli* K1 pathogens were determined by the gentamicin protection assay, which is based upon the principle that intracellular organisms are “protected” from the bactericidal effects of gentamicin, while extracellular organisms are killed. The invasion rate of E44 at the zero concentration of LGG was assigned as 100% and the effects of probiotic preincubation were compared to this control level (Figure 1(b)). As shown in Figure 1(b), LGG blocked E44 invasion of Caco-2 cells in a dose-dependent manner. The invasion ability of E44 was reduced by 78% at 1 × 10^8^ CFU of LGG (*P* < .01). A similar result was obtained with rat intestinal epithelial cell line IEC6 (data not shown).

### 3.2. Probiotics-Induced Blockage of Meningitic *E. coli* K1 Transcytosis Across the Intestinal Epithelial Barrier in vitro

**Figure 2:**
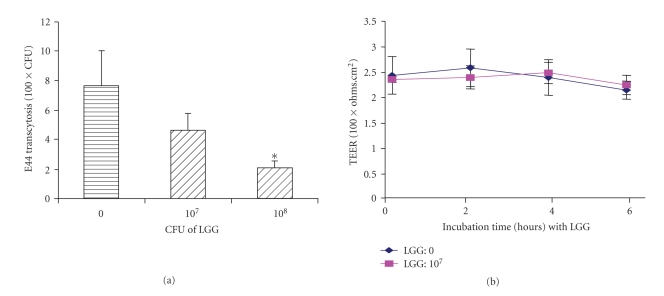
Effects of LGG on *E. coli* K1 translocation across Caco-2 monolayers (a) and transepithelial electrical resistance (TEER) of Caco-2 monolayers (b). (a) Epithelial cells were incubated with various doses of *L. rhamnosus* for 3 hours before adding 10^7^ E44. Transcytosis assays were carried out as described above. All values represent the means of triplicate determinations at 4 hours. Experiments were repeated three times. Error bars indicate standard deviations. *P < .05. (b) TEER was not significantly altered after 0 to 6 hours incubation.

The in vitro double chamber culture system in which cells are grown on porous filters has proven to be a valuable tool for the evaluation of bacterial transcytosis across the endothelial or epithelial barrier [25–27]. In order to examine whether LGG influences the internalized bacteria across the monolayers of Caco-2 cells using thetranscellular pathway with or without the enhancement of the epithelial barrier functions, competitive exclusion/transcytosis inhibition assays were carried out. In this experiment, Caco-2 cells were preincubated with different doses of LGG (10^7^ to 10^8^ CFU) before the addition of meningitic *E. coli* K1 strain E44. After incubation with LGG, 1 × 10^7^ CFU of E44 was added to the upper chamber of Transwell. The appearance of E44 in the bottom chamber was determined. Our studies suggested that LGG was able to significantly reduce transcytosis of E44 across the Caco-2 monolayers at 1 × 10^8^ CFU of LGG at 4 hours (*P* < .05) (Figure 2(a)). To further determine whether LGG influenced the barrier function that led to decreased E44 crossing the Caco-2 monolayers from the apical to the basolateral side the effect of LGG on the Caco-2 barrier function was evaluated by measuring the passage of HRP through confluent monolayers . HRP assay was carried out as previously described [30]. The HRP concentrationwas determined spectrophotometrically at 470 nm to determinethe peroxidase activity. E44 CFUs in the lower chamber were significantly reduced in the experiment group (E44 + LGG) at 1 × 10^8^ CFU of LGG compared to the control (E44 without adding LGG) (*P* < .05) (Figure 2(a)). However, stable TEER (Figure 2(b)) and HRP activity (25.6 ± 1.7 *μ*g/mL at 6 hours) were observed in both groups, suggesting that the barrier function or permeability was not remarkably altered.

### 3.3. Effects of LGG on Colonization, Bacteremia, and Meningitis of *E. coli* K1 in Neonatal Rats

The in vitro experiments demonstrated that the probiotic agent LGG was able to significantly block meningitic *E. coli* K1 adhesion, invasion, and transcytosis. Next, the probiotics-induced blocking effects on meningitic pathogens were further examined in the neonatal rat model of *E. coli* K1 meningitis. LGG was administrated orally to 2-day-old rats for 3 days before *E. coli* K1 infection. The 5-day-old rats were infected with *E. coli* E44, and the stool, blood, and CSF samples were cultured for indication of intestinal colonization, bacteremia, and meningitis, respectively. Our study showed that the rates of E44 intestinal colonization, bacteremia, and meningitis were significantly different between the experiment group with LGG and the control receiving PBS (Table 1). Quantitative cultures of LGG were also done with the blood samples from the pups receiving LGG. No LGG was detected. The average number of intestinal *E. coli* K1 colonies in the animals given LGG was significantly lower than that of the control group, suggesting that LGG is able to suppress *E. coli* K1 colonization in the rat intestine. No bacteremia and meningitis occurred in the whole animal group inoculated with LGG. In contrast, among the animals in the control group, 100% of animals colonized with meningitic *E. coli* K1 and the majority (64%) of the rats had bacteremia (from >10^5^ to 10^8^ CFU/mL), which is critical for the development of meningitis. Twenty one percent of the rats in the control group developed meningitis.

## 4. Discussion

In recent years, probiotic microorganisms have received increasing attention both from academics and from practitioners because of clinical observations suggesting that they are useful in preventing or treating some infectious diseases and allergic disorders [13–18, 31, 32]. These diseases include diarrhea, vaginitis, inflammatory bowel disease, and atopic dermatitis. Both prophylactic and therapeutic effects have been observed in both children and adults [32]. In the current studies, we have demonstrated for the first time that probiotics are able to suppress meningitic *E. coli* K1 penetration across intestinal epithelial cells in vitro and reduce bacteremia/meningitis in neonatal rats.

Bacterial adhesion and invasion are two subsequent steps essential for pathogen entry into the host cells. Enteric pathogens such as *E. coli* K1 must penetrate across two tissue barriers, the gut and the blood-brain barrier (BBB), in order to cause meningitis [1, 2]. *E. coli* K1 binding to and invasion of intestinal epithelial cells are a prerequisite for bacterial crossing of the gut barrier in vivo [33, 34]. In order to understand how probiotics suppress meningitic *E. coli* translocation through gastrointestinal epithelium, the present studies examined the effects of LGG on *E. coli* K1 strain E44 adhesion, invasion, and transcytosis in the human colon carcinoma cell line Caco-2, which is one of the most relevant in vitro models of gut epithelium for the studies of small intestinal epithelial cell differentiation, transport properties, and barrier functions [21–23, 33, 34]. The cells are fully differentiated after 14 days in culture, at which time they form a polarized monolayer with tight junctions and demonstrate dome formation, typical of transporting epithelial monolayers [21–23]. This in vitro cell culture model has been successfully used for identification of *E. coli* K1 S fimbria and *ibeA* as virulence factors required for efficient intestinal epithelial adhesion and invasion [23, 33]. The barrier integrity of the differentiated Caco-2 monolayers was assessed by TEER and HRP permeability. Stable TEER values and HRP activities were observed in both the control and treatment groups, suggesting that the barrier function or permeability was not remarkably altered. Our results show that in the in vitro Caco-2 cell line experiments LGG reduces *E. coli* K1 adhesion, invasion, and transcytosis.

To further assess the role of LGG inthe suppression of meningitic *E. coli* K1 infection, we conducted the animal study to test its biological functions using the newborn rat model of experimental hematogenous meningitis. This animal model of *E. coli* bacteremia and meningitis has been successfully established and used by us to assess the ability of pathogens to cross the gut barrier and the BBB in vivo [1, 2, 33]. Experimental *E. coli* bacteremia and meningitis in newborn murines have important similarities to human newborn *E. coli* infection, for example, age-dependency, hematogenous infection of meninges, without need for adjuvant or direct inoculation of bacteria into CSF [1, 2, 28]. The availability of this animal model enables us to examine the clinical relevance of probiotics-induced protective effects on newborns against the development of NSM. We showed that LGG was able to significantly reduce the pathogen intestinal colonization and the genesis of *E. coli* K1 bacteremia. LGG was not detected in the blood samples of the animals treated with the probiotics, suggesting that LGG, which has the most extensive safety assessment record and has never been the suspected causal agent of sepsis [35, 36], exhibited a high degree of safety in the neonatal murine pups. Twenty one percent of the animals in the control group had meningitis. No meningitis occurred in the rat pups treated with LGG. It has been previously shown that a high degree of bacteremia (>10^5^ bacteria/mL) is a primary determinant for meningeal invasion by *E. coli* K1 [2]. The rate of bacteremia in the animals treated with LGG (64%) was significantly lower than that of the control group (100%), indicating that the significantly decreased or even abolished translocation of the pathogen across the gut barrier led to a reduced number of bacteria or no bacteria entry into the bloodstream. This eventually resulted in no pathogens crossing the BBB to cause meningitis.

In conclusion, the results obtained in the current studies suggest that preventive administration of probiotic lactobacilli to infants may lead to enhanced resistance to neonatal bacterial sepsis and meningitis due to suppression of pathogen translocation across the gut barrier. Probiotics could be useful to correct ecological disorders in human intestinal microbiota associated with NSM and might play a protective role in excluding pathogens from the intestine and preventing infections.

## Figures and Tables

**Table 1:** The rates of *E. coli* colonization, bacteremia, and meningitis in rat pups after receiving E44 or E44 plus LGG.

Treatment	No. of animal	No. of pups with positive LGG culture in blood	No. (%) of pups with intestinal colonization (E44)	No. (%) of pups with bacteremia (10^5^ to 10^8^ CFU/mL) (E44)	No. (%) of pups with meningitis^a^
E44	14	—	14 (100)	9 (64)	3 (21)
E44 + LGG	14	0	8 (57)	0 (0)	0 (0)

^a^Defined as positive culture of CSF.
